# Wiedemann‐Steiner syndrome: A case report

**DOI:** 10.1002/ccr3.3704

**Published:** 2021-01-05

**Authors:** Lorna Hirst, Robert Evans

**Affiliations:** ^1^ Dental and Maxillofacial Department Great Ormond Street Hospital London UK

**Keywords:** dentistry, genetics, pediatric and adolescent medicine

## Abstract

Wiedemann‐Steiner syndrome (WDSTS) is an exceptionally rare autosomal dominant syndrome with considerable phenotypical variation. Clinical features include dysmorphic facial and skeletal features, growth deficiency, developmental delay, hypertrichosis cubiti and various dental features. We present a 7‐year‐old female with premature exfoliation of primary teeth and premature eruption of permanent teeth.

## Graphical abstract

Premature exfoliation of primary teeth is a significant diagnosis that necessities further investigation. Differential diagnoses are vast, comprising of multisystemic disorders, and life‐threatening illnesses, such as hematological malignancies.

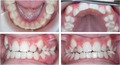

## 1 INTRODUCTION

Wiedemann‐Steiner syndrome (WDSTS, OMIM #605130)^1^ is an exceptionally rare autosomal dominant syndrome with a prevalence of <1 in 1 000 000.^1^ Considerable phenotypical variation exists,^2^ with clinical characteristics encompassing dysmorphic facial and skeletal features, pre‐ and postnatal growth deficiency, developmental delay, and hypertrichosis cubiti.^3^, ^4^, ^5^ The dental findings previously described include, premature exfoliation of primary teeth, premature eruption of permanent teeth, hypodontia, widely spaced dentition, malocclusion, supernumerary teeth, and cleft palate.^5^, ^6^ The rarity of WDTS presents a challenge in describing the complete phenotypic spectrum.

WDSTS was initially reported by Wiedemann in 1989 and is classified as a chromatin remodeling defect.^3^ Genetically, whole exome sequencing in 2012 identified the etiology as heterogeneous de novo mutations in the KMT2A (lysine methyltransferase) gene.^2^, ^4^, ^5^ KMT2A encodes a histone methyltransferase, an enzyme important in the regulation of chromatin‐mediated transcription.^2^, ^4^

Failure to thrive presents a nonspecific, but prominent feature in WDSTS patients.^5^ Early neuropsychiatric indications comprise difficulties sleeping, aggressive behavior, hyperactivity, autistic‐like qualities, and a broad range of intellectual disability and developmental delay.^4^, ^5^ Facial dysmorphic features generally become more noticeable with age and comprise down‐slanting palpebral fissures, telecanthus, strabismus, synophrys, long eyelashes, wide nasal bridge, thin upper lip, long philtrum, and high arched palate.^5^, ^7^, ^8^

This case outlines a seven‐year‐old girl, with a confirmed diagnosis of WDSTS, who presented with a history of the exfoliation of all primary teeth between 24 and 36 months and commencement of the eruption of permanent dentition aged four.

## 2 CASE REPORT

**FIGURE 1:**
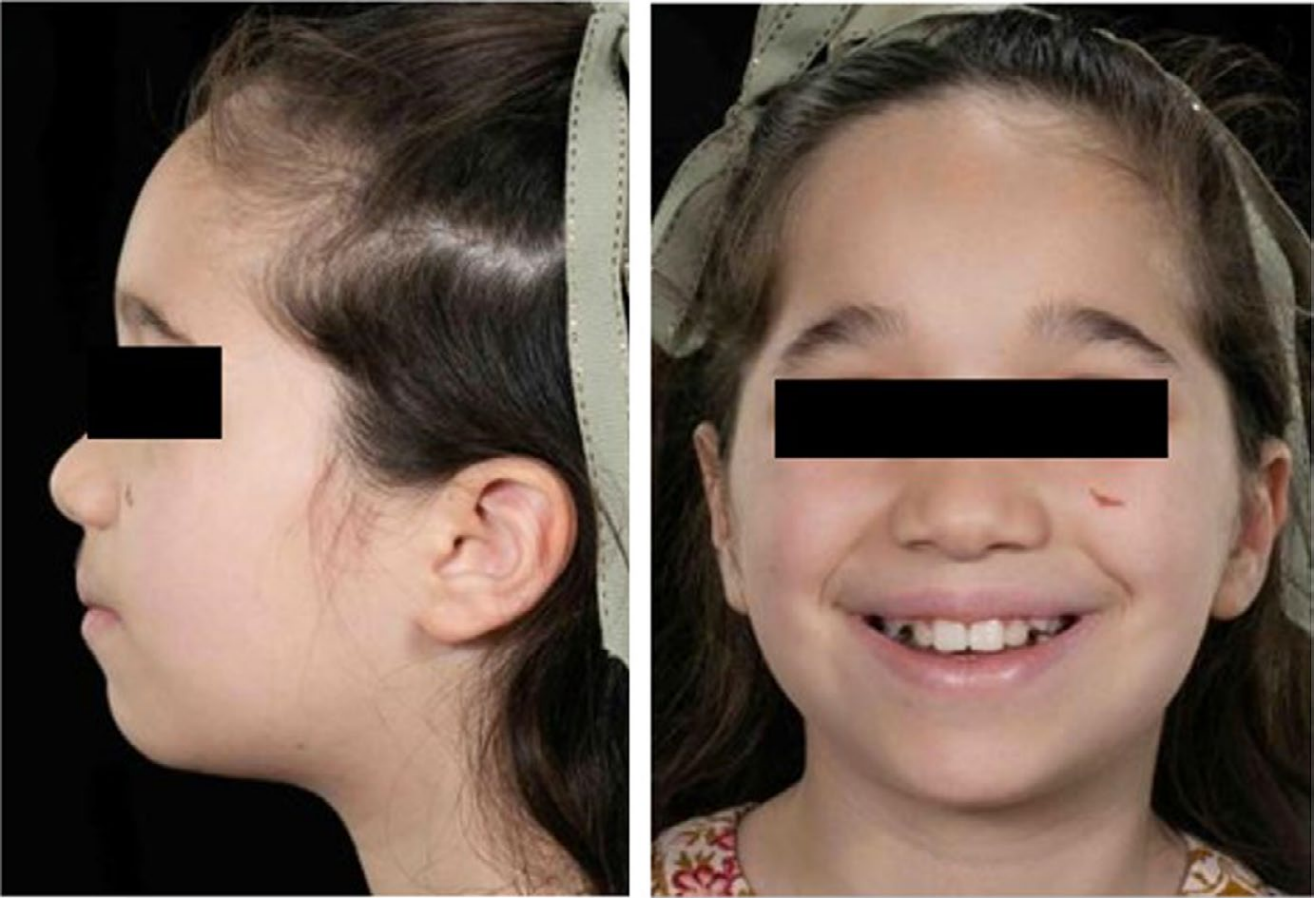
Facial views showing mild dysmorphic facial features, taken age seven

The patient is the oldest of three siblings. Her younger brother and sister do not have WDSTS. She was born at term following an uneventful pregnancy at a birth weight of 3.32 kg (25th centile). Feeding difficulties were experienced from birth requiring a tongue tie release. An assessment by a clinical geneticist aged 5 years, 10 months old, showed several distinctive facial features including telecanthus, flared medial eyebrows, long and narrow palpebral fissures, a long smooth philtrum, and bilateral ear pits (Figure 1). Generalized hypertrichosis was present as well as a sacral dimple. There was a history of bilateral hip dysplasia requiring varus de‐rotation osteotomies of the proximal femora. Ophthalmological input was arranged for intermittent exotropia and a right convergent squint which self‐corrected. She had global developmental delay and her parents expressed concerns of autistic spectrum features such as resistance to changes in routine, educational difficulties, and sensory issues including textures. Subsequent testing for the Cornelia de Lange syndrome panel of genes revealed a heterozygous pathogenic variant c.3082A>T, p.(Lys1028Ter) in the KMT2A gene, confirming the diagnosis of WDSTS.

At age 6 years and 9 months, an orthodontic assessment was carried out. This revealed that all her primary teeth had erupted by eight months and that most of the primary teeth had exfoliated between the ages of 24‐36 months. Her oral hygiene was fair, and no caries were present. The enamel appeared normal. The teeth present were:

6E4321/1234E6

654321/1234E6

**FIGURE 2:**
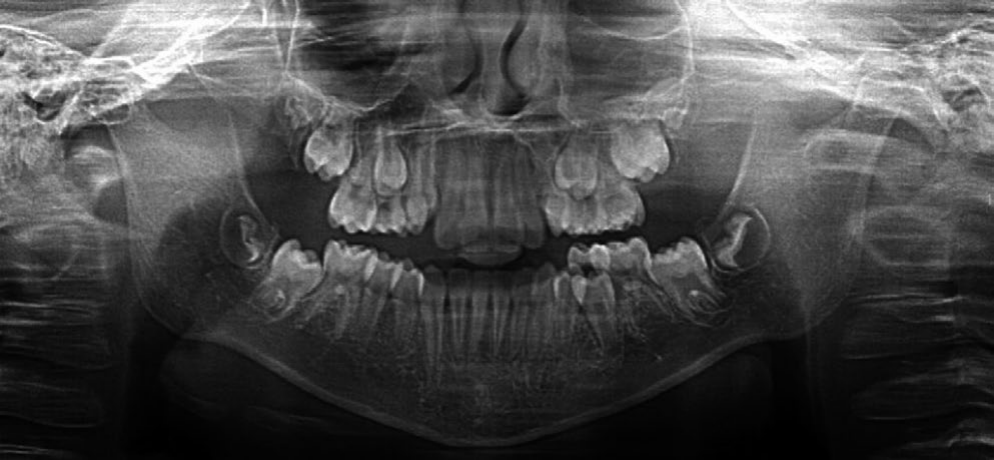
Orthopantomogram (OPG) taken at age seven years old

**FIGURE 3:**
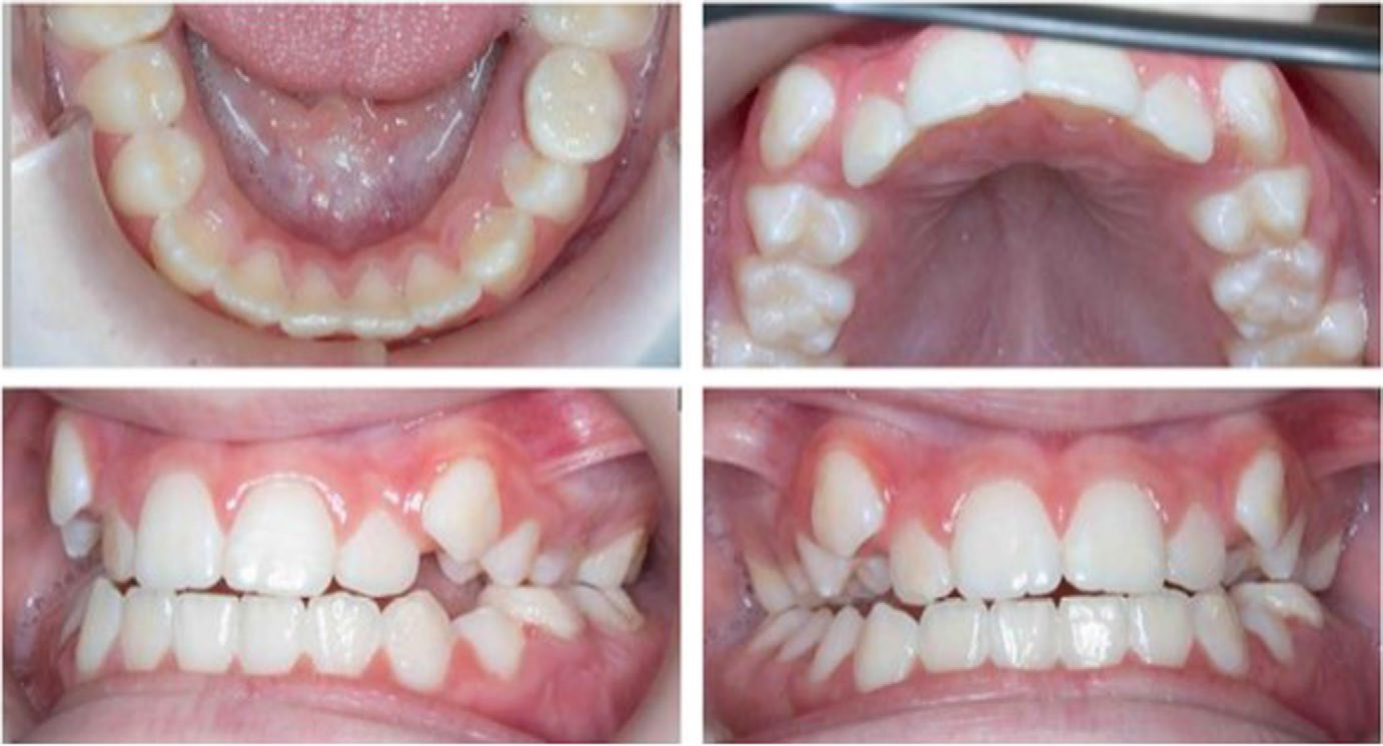
Intraoral photographs aged seven

An orthopantomogram (OPG) was taken (Figure 2). This showed that all the permanent teeth were developing including all four third permanent molars. The clinical examination showed a mild Class III incisor relationship (edge to edge) and buccal displacement of both the erupting maxillary permanent canines with mild crowding (Figure 3). The palate was normal.

## 3 DISCUSSION

WDSTS is a very rare autosomal dominant disorder with relatively few cases described in the literature. Considerable phenotypical variation has been described, which may increase as additional patients are diagnosed. Multiple anomaly syndromes such as Kabuki, Rubenstein Taybi, Pierpont, and Cornelia De Lange overlap phenotypically with WDSTS creating a diagnostic challenge. Etiologically, a substantial number of these genetic syndromes involve pathogenic variants in genes encoding histone modification and chromatin remodeling.^3^

The literature describes several oral and dental anomalies in patients with WDSTS (Table 1), with premature eruption of primary and permanent teeth being commonly cited.^5^, ^6^, ^9^, ^10^

**TABLE 1:** Dental abnormalities in WDSTS.^5^, ^6^, ^9^, ^10^

Premature eruption of dentition
Premature exfoliation of primary dentition
Hypodontia
Dental spacing
Malocclusion
Cleft palate
Retrognathia
Supernumerary teeth
High arched palate
Glossoptosis

The premature exfoliation of primary dentition and/or the premature eruption of permanent dentition can be attributed to local, environmental, or systemic factors or be considered as a mild nonpathological variation from normal.^11^ While it is very unlikely that a patient with WDSTS would see a pediatric dentist without a confirmed diagnosis, it is important to be aware of this and other medical conditions which affect the usual dates for tooth eruption and exfoliation, for both primary and secondary dentition. The conditions listed in Table 2 are known to present with the premature exfoliation of the primary teeth and/or premature eruption of the permanent dentition.

**TABLE 2:** Commonly cited systemic disorders associated with premature eruption of secondary teeth and premature exfoliation of primary teeth, presented from the most to the least common^11^, ^12^, ^13^, ^14^, ^15^, ^16^, ^17^, ^18^, ^19^, ^20^, ^21^

Diagnosis	Classification	Common clinical features
Premature exfoliation of primary teeth		
Rare disorders		
Acute Lymphoblastic Leukemia Prevalence: 11.0/100 000^a^ Acute Myeloid Leukemia Prevalence: 10.0/100 000^a^	Hematological Malignancy	Gingival Hemorrhage and Hyperplasia, Petechiae, Periodontal Disease, Lymphadenopathy
Ehlers‐Danlos Syndrome Prevalence: 5.0/100 000	Connective Tissue Disorder	Periodontal Disease, Fragile Skin, Bruising of Skin, Muscle Fatigue and Pain, Loose Joints.
Acatalasia Prevalence: 3.2/100 000^a^	Metabolic Disorder	Oral Ulceration, Gangrene
Ultra‐Rare Disorders		
Coffin‐Lowry Syndrome Prevalence: 1.5/100 000	Genetic Multisystem Disorder	Cardiac Abnormalities, Kyphoscoliosis, Short Stature, Cognitive Difficulties
Langerhans Cell Histiocytosis Prevalence: 1.5/100 000^a^	Hematological Disorder / Malignancy	Single / Multiple Lytic Bone Lesions, Gingival Inflammation, Drifting of Teeth, Ulceration, Bone Destruction
Erythromelalgia Prevalence: 1.0/100 000	Neurovascular Disorder	Erythema / Swelling of Soft Tissue, Burning Sensation of Extremities
Papillon‐Lefèvre Syndrome Prevalence: 0.25/100 000	Dermatological Disorder	Hyperkeratosis of Palms and Soles of Feet, Early Onset Periodontal Disease
Hypophosphatasia Birth Prevalence: 0.21/100 000^a^	Metabolic Bone Disease	Taurodontism, Hypoplastic or Absent Cementum, Skeletal Deformities, Widened Fontanelles.
Chediak Higashi Syndrome Documented Cases: 500	Genetic Immunological Disorder	Recurrent and Persistent Infections, Albinism, Strabismus, Nystagmus, Periodontal Destruction
Leukocyte Adhesion Deficiency Documented Cases: 350	Immunological Disorder	Recurrent Bacterial Infections, Gingival Inflammation and Alveolar Bone Loss.
Hajdu‐Cheney Syndrome (Acroosteolysis) Documented Cases: 100	Connective Tissue Disorder	Osteoporosis, Short Stature, Skull and Facial Abnormalities.
Singleton Merten Syndrome Documented Cases: 22	Genetic Multisystem Disorder	Calcification of Aortic Arch, Glaucoma, Photosensitivity
Premature Eruption of Permanent Dentition		
Commoner Diseases		
Hyperthyroidism Prevalence: 81.0/100 000	Endocrinological Disorder	Heat Intolerance, Weight Loss, Hand Tremor, Irritability, Sleeplessness, Tachycardia
Type 1 Diabetes Mellitus Prevalence: 59.0/100 000	Endocrinological Disorder	Polyuria, Polydipsia, Polyphagia
Rare Diseases		
Soto Syndrome Birth Prevalence: 7.1/100 000	Congenital, Genetic Overgrowth Disorder	Overgrowth, Facial Deformities, Advanced Bone Age, Developmental Issues, Cardiovascular, Ophthalmological and Urogenital Manifestations
Congenital Adrenal Hyperplasia Prevalence: 7.0/100 000^a^	Congenital Endocrinological Disorder	Premature Adrenarche, Advanced Bone Age, Accelerated Growth. Pubertal Disturbances
Turner Syndrome Birth Prevalence: 5.5/100 000^a^	Genetic Endocrinological Disorder	Short Stature, Gonadal Dysgenesis, Lymphoedema, Micrognathia
Sturge‐Weber Syndrome (Encephalotrigeminal Angiomatosis) Birth Prevalence: 3.5/100 000^a^	Congenital Neurocutaneous Syndrome	Cutaneous Vascular Malformations; Unilateral and Facial
Ultra‐Rare Diseases		
Chondroectodermal Dysplasia (Ellis‐Van Creveld Syndrome) Birth Prevalence: 1.1/100 000	Genetic Skeletal Dysplasia	Ectodermal Dysplasia, Cardiac Defects, Disproportionate Dwarfism
Klippel–Trénaunay Syndrome Prevalence: 0.007/100 000^a^	Congenital Vascular Disorder	Unilateral Soft Tissue and Bony Hypertrophy, Hemifacial Hypertrophy, Hemangiomas, Varicosities
Pachyonychia Congenita Documented Cases: 1000	Genetic Dermatological Disorder	Hyperkeratosis of Palms and Soles, Paronychial Infections, Corneal Dyskeratosis, Alopecia, Hyperhidrosis.
Hemihyperplasia‐multiple Lipomatosis Syndrome Documented Cases: 10	Genetic Overgrowth Syndrome	Craniofacial Deformities, Ipsilateral Macroglossia, Macrodontia

^a^ European Data.

The eruption of the permanent teeth in advance of the expected time means that orthodontic treatment can commence earlier than usual. For this patient, the plan is to use, in the first instance, a fixed palatal arch to maintain the leeway space and then once both maxillary second premolars (UR5, UL5) have erupted, fixed appliances to align the teeth and correct the incisor relationship. What is not known at this stage is the extent and direction of future facial growth which could affect the outcome.

For patients with WDSTS, an early referral to an orthodontist is recommended to compliment the pediatric dental care.

## CONFLICT OF INTEREST

None declared.

## AUTHOR CONTRIBUTION

LH: wrote the first draft of the manuscript and revised subsequent drafts. RE: selected the case and reviewed, edited, and provided guidance for subsequent drafts of the manuscript.

## ETHICAL APPROVAL

Informed written consent was obtained from the parents in this case report prior to submission regarding the publication of images and data.

## Data Availability

Data sharing was not applicable to this article as no datasets were generated or analyzed in the production of the manuscript.
